# Greater mastery is associated with lower depression risk in a large international cohort of people with multiple sclerosis over 2.5 years

**DOI:** 10.1007/s11136-021-03033-7

**Published:** 2021-11-23

**Authors:** Sandra Neate, Afaf Humam, Nupur Nag, George A. Jelinek, Steve Simpson-Yap

**Affiliations:** 1 Neuroepidemiology Unit, Melbourne School of Population and Global Health, The University of Melbourne, 207 Bouverie St, Carlton, VIC 3053 Australia; 2 Menzies Institute for Medical Research, University of Tasmania, Hobart, Australia

**Keywords:** Multiple sclerosis, Mastery, Depression, Disability, Fatigue, Relapse, Cohort, Epidemiology

## Abstract

**Background:**

Mastery is the extent to which an individual perceives their life circumstances as being under their control and not predominantly influenced by external factors. The relationship of mastery with clinical outcomes in people with multiple sclerosis (pwMS) has not been well-researched. We assessed the relationships of mastery with fatigue, disability, relapse number, and depression risk among pwMS over 2.5 years’ follow-up.

**Methods:**

Data from the Health Outcomes and Lifestyle in a Sample of people with Multiple sclerosis study, among 839 participants who completed the 2.5 and 5-year reviews, were analysed. Mastery was measured by the Pearlin Mastery Scale, fatigue by Fatigue Severity Scale, depression risk by Patient Health Questionnaire-9, and disability by Patient-Determined Disease Steps, and diagnosed relapse number in the previous 12 months was queried. Cross-sectional and prospective analyses were undertaken by log-binomial, log-multinomial, and Poisson regression, as appropriate, adjusted for relevant confounders.

**Results:**

Cross-sectionally, pwMS with the highest quartile mastery (> 25/28) had 90% lower frequency of depression risk, 60% lower frequency of clinically significant fatigue, and 77% fewer had severe disability, all largely robust to adjustment. Prospectively, those in the top two quartiles of mastery (> 21–25, > 25/28) had 66% and 74% lower subsequent depression risk, robust to adjustment. No significant associations were seen prospectively for change in fatigue, disability, or relapse number, however, and no robust associations of mastery with relapse number were evident.

**Conclusions:**

Prospectively, a protective relationship of mastery with subsequent risk of depression was observed, suggesting this may be a point of intervention to improve wellbeing in pwMS.

**Supplementary Information:**

The online version contains supplementary material available at 10.1007/s11136-021-03033-7.

## Introduction

Multiple sclerosis (MS) is an autoimmune neurodegenerative disease that affects the central nervous system. The multiple physical, emotional, and social challenges faced by people with MS (pwMS) have a significant impact on feelings of control over their lives. The sense of autonomy and agency, that is, the capacity to act independently and make one’s own choices, may be impaired by the impact of these challenges. When faced with stressful life events or challenges, people draw upon their inner psychological resources and characteristics. These resources can be extremely effective at assisting in developing coping mechanisms. Pearlin described psychological resources involved in coping including self-esteem, defined as the positiveness of one’s attitude towards oneself, and mastery, the extent to which one regards one’s life-chances as being under one's own control in contrast to being fatalistically ruled [1]. Self-efficacy is the expectation and confidence that the person has of their ability to employ the described psychological resource to affect an outcome [2]. Self-efficacy is part of a theoretical framework for analysing the outcomes or achievements of employing psychological resources to cope or reduce stress, of which mastery is one [2].

Studies examining self-esteem in pwMS have identified that improved self-esteem is associated with the use of strategies for improved coping and that feelings of self-worth are linked to the ability to handle challenging life situations [3]. Self-efficacy in MS has been identified as a determinant of several outcomes including perceived health status [4, 5], ability to adjust to illness [6], and health-related quality of life [7]. Mastery has also been associated with a dose-dependent improvement in both mental and physical health-related quality of life in pwMS [8].

With respect to specific health outcomes in MS, an improved sense of mastery has been demonstrated to ameliorate fatigue, the distress associated with fatigue, and the subsequent inability to perform tasks [9]. The treatment of depression in MS has been shown to improve the individual’s sense of mastery, sense of purpose in life, and their level of self-acceptance [10]. However, the impact of mastery on other clinical outcomes in PwMS has not been well-explored. This study therefore assessed the cross-sectional and prospective relationships of mastery with fatigue, level of disability, relapse number, and depression risk over 2.5 years in an international cohort of pwMS.

## Methods

### Participants

Data from the Health Outcomes and Lifestyle In a Sample of people with Multiple sclerosis (HOLISM) study were analysed. The methodology of the HOLISM study has been described previously [11, 12]. Briefly, participants were recruited through Web 2.0 platforms and invited to complete an online survey. Consenting individuals at least 18 years of age proceeded to the survey and were subsequently invited to complete follow-up surveys at 2.5-year intervals thereafter. All analyses are constrained to persons reporting a physician diagnosis of MS at baseline. Here, we report analyses of data from the 2.5-year timepoint (referred to as the 2.5-year review) when mastery was first queried and follow-up 2.5 years later, that is 5 years from the baseline survey (referred to as the 5-year review).

Ethics approval was given by The University of Melbourne’s Health Sciences and Human Ethics Sub-Committee (HESC 1545102).

### Data collection and tools

Participants completed a multi-item questionnaire capturing demographic, lifestyle, and clinical characteristics. Data included age, sex, marital status, country of residence, level of education, perceived relative socioeconomic status (SES) [13], height and weight from which body mass index (BMI) was calculated, physical activity (International Physical Activity Questionnaire, IPAQ [14]), number of people in their core social support network (queried as “How many people do you have near you that you can readily count on for help in times of difficulty, such as watch over children or pets, give rides to hospital or store, or help when you are sick”), MS type, baseline number of treated comorbidities[15], and medication use, as described previously [12].

The primary exposure of interest was mastery, measured by the Pearlin Mastery Scale (PMS) [1]. The scale consists of seven statements, each measured on a 4-point Likert scale, where 1 equals strongly agree and four strongly disagree. Responses were summated to realise a total PMS score ranging from 7 (low mastery) to 28 (high mastery), and then categorised into quartiles.

Four clinical outcomes were assessed at each timepoint by validated tools. Fatigue was assessed by the Fatigue Severity Scale (FSS) [16], where clinically significant fatigue was defined as mean FSS score > 5. Disability was measured using the Patient-Determined Disease Steps (PDDS) [17], and categorised as mild (PDSS = 0–3), moderate (PDSS > 3–6), and severe (PDSS > 6). Depression risk was assessed by the Patient Health Questionnaire-9 (PHQ-9) [18], a score > 9 indicating depression risk. Doctor-diagnosed relapse number in the previous 12 months was queried. Participants were also asked whether they were experiencing ongoing symptoms due to recent relapse in the preceding 30 days.

All measures were obtained at each timepoint, except for mastery and perceived relative SES which were only measured from 2.5-year onwards, while MS type, age, and comorbidity number were measured at baseline.

### Data analysis

Characteristics of mastery at 2.5-year review were assessed by linear regression, adjusted for ongoing symptoms from recent relapse, and further adjusted for age, sex, education, support number, PDDS, clinically significant fatigue, depression risk, and baseline treated comorbidity number. For cross-sectional analyses at 2.5-year review, clinically significant fatigue, disability, and depression risk were assessed by log-binomial regression, and relapse number by Poisson regression. All models except for relapse number were adjusted at minimum for whether participants were experiencing ongoing symptoms from recent relapse. Full multivariable models were constructed and covariates that materially affected models developed, these including age, sex, MS type, PDDS, clinically significant fatigue, depression risk, and antidepressant medication use, as appropriate for the outcome.

For change analyses from the 2.5 to 5-year reviews, dichotomous outcomes (fatigue, depression risk) were evaluated by log-binomial regression, as the proportion of pwMS without the outcome at either timepoint compared with the subset who developed the outcome at 5-year review (gain of outcome), and the proportion of pwMS with the outcome at both timepoints compared with the subset who did not have that outcome at 5-year review (loss of outcome), a method we have used previously [19]. Change in disability and relapse number were evaluated as a difference in the outcome, with “increasing”, “decreasing” or “the same” evaluated as polychotomous outcomes assessed by log-multinomial regression. For disability, fatigue, and depression analyses, all models were adjusted for the 2.5-year values of the outcome variable, as well as whether participants were experiencing ongoing symptoms from recent relapse at 2.5- and 5-year reviews.

For continuous terms, evaluation of homoscedasticity of model residuals were undertaken and normality sufficient for linear regression verified.

All analyses were conducted using Stata SE/15.0 (StataCorp, College Station, TX, USA).

## Results

Of 2466 baseline participants, 1401 (58.4%) completed the survey at 2.5-year review. Of the 1401 participants, 839 (59.9%) also completed the 5-year review and are included in this analyses.

Of the 839 participants, *n* = 793 (94.5%) completed the Pearlin Mastery Scale questions. Respondents to the PMS were mostly female (82.4%), had a mean age of 51 years, and 64.2% had between two and five support people in their lives. The largest proportion of participants lived in Australia/New Zealand (47.3%), most had completed university (69.8%), and were of higher perceived socioeconomic status (54.0%). Respondents were predominantly diagnosed with benign or relapsing–remitting MS (RRMS; 70.7%), were of normal BMI (64.1%), and reported no treated comorbidities (62.4%). The average disease duration from onset at 5-year review was 19.0 years (SD = 10.1).

Mastery was significantly higher among those with postgraduate degrees, higher SES, more than six members in their social support network, and those who were physically active, while mastery was lower among those of progressive MS type, severe disability, with clinically significant fatigue, and with depression risk, all robust to adjustment for age, sex, education, support number, PDDS, clinically significant fatigue, depression risk, and baseline comorbidity number. Other characteristics of mastery are shown in Table 1.

**Table 1:** Characteristics of mastery at 2.5-year review

Characteristics	n/N (%)	*aβ* (95% CI)^a^	*aβ* (95% CI)^b^
Sex			
Male	140/793 (17.7%)	0.00 [Reference]	0.00 [Reference]
Female	653/793 (82.4%)	− 0.19 (− 0.95, 0.57) *p* = *0.62*	0.21 (− 0.49, 0.92) *p* = *0.55*
Age			
18.0–42.9	202/793 (25.5%)	0.00 [Reference]	0.00 [Reference]
> 42.9–51.0	198/793 (25.0%)	0.11 (− 0.71, 0.93)	0.41 (− 0.34, 1.16)
> 51.0–58.5	201/793 (25.4%)	− 0.11 (− 0.92, 0.70)	0.62 (− 0.15, 1.39)
> 58.5	192/793 (24.2%)	− 0.55 (− 1.38, 0.28)	0.51 (− 0.30, 1.31)
*Trend:*		*p* = *0.17*	*p* = *0.14*
Education level			
Up to secondary	122 (15.4%)	0.00 [Reference]	0.00 [Reference]
Vocational training	117 (14.8%)	**1.29 (0.24, 2.33)**	**1.15 (0.19, 2.11)**
Bachelor’s degree	305 (38.5%)	**1.57 (0.70, 2.43)**	**1.06 (0.24, 1.88)**
Post-graduate degree	248 (31.3%)	**2.00 (1.10, 2.89)**	**1.46 (0.62, 2.30)**
*Trend:*		***p* < *0.001***	***p* = *0.003***
Perceived relative socioeconomic status			
Lower	130/789 (16.5%)	**− 1.49 (− 2.32, − 0.66)**	0.05 (− 0.78, 0.89)
Same	233/789 (29.5%)	0.00 [Reference]	0.00 [Reference]
Higher	426/789 (54.0%)	**1.18 (0.54, 1.81)**	**0.94 (0.30, 1.59)**
*Trend:*		***p* < *0.001***	***p* = *0.006***
Number of people in core social support network			
0	19/770 (2.5%)	0.00 [Reference]	0.00 [Reference]
1	157/770 (20.4%)	0.63 (− 1.35, 2.60)	0.89 (− 0.84, 2.61)
2–5	494/770 (64.2%)	1.52 (− 0.38, 3.42)	1.21 (− 0.45, 2.87)
6–9	61/770 (7.9%)	**2.88 (0.75, 5.02)**	**2.25 (0.37, 4.13)**
10 +	39/770 (5.1%)	**3.07 (0.80, 5.34)**	**2.65 (0.66, 4.65)**
*Trend:*		***p* < *0.001***	***p* < *0.001***
BMI			
Normal (> 18.5–25)	508/793 (64.1%)	0.00 [Reference]	0.00 [Reference]
Overweight (> 25–30)	164/793 (20.7%)	**− 1.18 (− 1.91, − 0.46)**	− 0.37 (− 1.05, 0.30)
Obese (> 30)	121/793 (15.3%)	**− 1.16 (− 1.98, − 0.34)**	0.38 (− 0.44, 1.20)
*Trend:*		***p* < *0.001***	*p* = *0.70*
IPAQ			
Inactive	222/778 (28.5%)	0.00 [Reference]	0.00 [Reference]
Minimally active	352/778 (45.1%)	**2.03 (1.36, 2.71)**	**0.82 (0.15, 1.50)**
Active	205/778 (26.4%)	**2.60 (1.84, 3.36)**	**1.04 (0.26, 1.82)**
*Trend:*		***p* < *0.001***	***p* = *0.010***
MS type			
Benign/RRMS	577/788 (70.7%)	0.00 [Reference]	0.00 [Reference]
SPMS/PPMS/PRMS	136/788 (17.1%)	**− 1.83 (− 2.60, − 1.05)**	− 0.27 (− 1.18, 0.65)
Unsure/Other	96/788 (12.2%)	− 0.30 (− 1.20, 0.59)	− 0.26 (− 1.09, 0.58)
Duration since MS onset, years			
2.88–8.01	198 (25.0%)	0.00 [Reference]	0.00 [Reference]
> 8.01–13.92	204 (25.8%)	− 0.39 (− 1.21, 0.42)	− 0.07 (− 0.83, 0.68)
> 13.92–22.22	197 (24.9%)	− 0.72 (− 1.54, 0.10)	0.03 (− 0.78, 0.84)
> 22.22–53.92	193 (24.4%)	**− 1.26 (− 2.08, − 0.43)**	0.15 (− 0.74, 1.03)
*Trend:*		***p* = *0.002***	*p* = *0.72*
PDDS			
Normal/mild	486 (61.4%)	0.00 [Reference]	0.00 [Reference]
Moderate	226 (28.5%)	**− 1.80 (− 2.43, − 1.16)**	**− 0.75 (− 1.40, − 0.10)**
Severe	80 (10.1%)	**− 2.81 (− 3.76, − 1.85)**	**− 1.56 (− 2.51, − 0.61)**
*Trend:*		***p* < *0.001***	***p* < *0.001***
Clinically significant fatigue			
No	319/759 (42.0%)	0.00 [Reference]	0.00 [Reference]
Yes	440/759 (58.0%)	**− 3.39 (− 3.94, − 2.83)** ***p* < *0.001***	**− 2.08 (− 2.69, − 1.48)** ***p* < *0.001***
MS immunomodulatory medication use			
None	444/793 (56.0%)	0.00 [Reference]	0.00 [Reference]
Interferon-β	77/793 (9.7%)	− 0.46 (− 1.47, 0.54)	− 0.64 (− 1.58, 0.29)
Other^c^	272/793 (34.3%)	− 0.51 (− 1.14, 0.12)	− 0.46 (− 1.06, 0.13)
Depression risk (PHQ-9)			
No	622/762 (81.6%)	0.00 [Reference]	0.00 [Reference]
Yes	140/762 (18.4%)	**− 4.34 (− 5.03, − 3.64)** ***p* < *0.001***	**− 2.92 (− 3.64, − 2.19)** ***p* < *0.001***
Number of treated comorbidities			
0	495/793 (62.4%)	0.00 [Reference]	0.00 [Reference]
1	174/793 (21.9%)	**− 1.23 (− 1.93, − 0.52)**	− 0.51 (− 1.17, 0.16)
2	88/793 (11.1%)	**− 1.86 (− 2.79, − 0.94)**	− 0.68 (− 1.58, 0.22)
3 or more	36/793 (4.5%)	**− 2.84 (− 4.22, − 1.46)**	− 1.31 (− 2.64, 0.02)
*Trend:*		***p* < *0.001***	***p* = *0.013***
Prescription antidepressant medication use			
No	671 (84.6%)	0.00 [Reference]	0.00 [Reference]
Yes	122 (15.4%)	**− 2.40 (− 3.19, − 1.62)** ***p* < *0.001***	− 0.12 (− 0.97, 0.73) *p* = *0.78*

All models by linear regression, estimating *β* (95% CI). Results in boldface denote statistical significance (*p* < 0.05)

*BMI* Body mass index; *IPAQ* International physical activity questionnaire; *PHQ-9* Patient health questionnaire 9; *PDD* Patient determined disease steps; *PPMS* Primary progressive MS; *PRMS* Progressive-relapsing MS; *RRMS* Relapsing–remitting MS; *SPMS* Secondary-progressive MS

^a^Multivariable model adjusted for ongoing symptoms of recent relapse

^b^Multivariable model adjusted for ongoing symptoms of recent relapse and further adjusted for age, sex, education, support number, PDDS, clinically significant fatigue, depression risk, and treated comorbidity number

^c^Other DMTs include glatiramer acetate, alemtuzumab, cladribine, daclizumab, dimethyl fumarate, fingolimod, laquinimod, rituximab, teriflunomide, and natalizumab

### Cross-sectional analyses of outcomes at 2.5-year review

#### Depression risk

A dose-dependent inverse association between mastery and depression risk was found, persisting upon adjustment for age, sex, clinically significant fatigue, disability, baseline number of treated comorbidities, and prescription antidepressant medication use (Supplemental Table 1). Participants with the highest quartile higher mastery score had 90% lower frequency of depression risk compared to those the lowest mastery score.

#### Fatigue

The majority (82.8%) of participants with a mastery score of 7–19 had clinically significant fatigue (Supplemental Table 1). FSS and mastery were inversely associated, persisting on adjustment for ongoing symptoms of recent relapse, age, sex, disability, depression risk, and baseline number of treated comorbidities, and showing a dose-dependent association. Participants scoring in the top two quartiles of mastery had 31% and 50%, respectively, lower frequencies of clinically significant fatigue.

#### Relapse number

In univariable analyses, those with higher mastery score had significantly fewer relapses in the preceding 12 months (Supplemental Table 1). On adjustment for age, sex, clinically significant fatigue, disability, depression risk, and baseline number of treated comorbidities, however, this association attenuated and lost dose-dependency.

#### Disability

An inverse association was found between mastery and both moderate and severe disability (Supplemental Table 2). On adjustment for age, sex, clinically significant fatigue, depression risk, and baseline number of treated comorbidities, however, only the association of mastery with severe disability persisted.

### Prospective change analyses

#### Change in depression risk

Mastery above the median was associated with significantly lower risk of developing depression risk, this attenuating only slightly on adjustment such that those in the top two quartiles had 63% and 68% lower risk of developing depression risk, respectively (Table 2). Consolidating the top and bottom two quartiles found that those with mastery above the median had 70% lower risk of developing depression risk, persisting on adjustment. No association with losing depression risk was seen, however.

**Table 2:** Mastery and change in depression risk

	*n* (row %)		Loss of depression risk		*n* (row %)		Gain of depression risk	
	Always depression risk	Stops depression risk	aRR (95% CI)^a^	aRR (95% CI)^b^	Never depression risk	Develops depression risk	aRR (95% CI)^a^	aRR (95% CI)^b^
Mastery								
7–19	53 (58.9%)	37 (41.1%)	1.00 [Reference]	1.00 [Reference]	107 (83.0%)	22 (17.1%)	1.00 [Reference]	1.00 [Reference]
> 19–21	14 (56.0%)	11 (44.0%)	1.05 (0.63, 1.75)	1.04 (0.66, 1.66)	100 (83.3%)	20 (16.7%)	0.98 (0.56, 1.71)	0.99 (0.55, 1.77)
> 21–25	10 (66.7%)	5 (33.3%)	0.80 (0.38, 1.67)	0.90 (0.43, 1.87)	181 (94.3%)	11 (5.7%)	**0.34 (0.17, 0.67)**	**0.37 (0.18, 0.75)**
> 25–28	4 (57.1%)	3 (42.9%)	0.99 (0.43, 2.29)	1.20 (0.43, 3.38)	156 (95.7%)	7 (4.3%)	**0.26 (0.11, 0.58)**	**0.32 (0.14, 0.75)**
*Trend:*			*p* = *0.75*	*p* = *0.85*			***p* < *0.001***	***p* = *0.001***
Mastery								
7–21	67 (58.3%)	48 (41.7%)	1.00 [Reference]	1.00 [Reference]	207 (83.1%)	42 (16.9%)	1.00 [Reference]	1.00 [Reference]
> 21–28	14 (63.6%)	8 (36.4%)	0.85 (0.48, 1.52) *p* = *0.58*	0.98 (0.53, 1.80) *p* = *0.95*	337 (94.9%)	18 (5.1%)	**0.30 (0.18, 0.51)** ***p* < *0.001***	**0.35 (0.21, 0.60)** ***p* < *0.001***

All analyses by log-multinomial regression, estimating (aRR (95% CI). Results in boldface denote statistical significance (p<0.05)

^a^Multivariable log-binomial regression model adjusted for baseline depression risk (PHQ-9), baseline ongoing symptoms of relapse and ongoing symptoms of relapse

^b^Multivariable log-binomial regression model adjusted for baseline depression risk (PHQ-9), age, sex, baseline disability, number of treated comorbidities, baseline clinically significant fatigue, and prescription antidepressant medication use

#### Change in clinically significant fatigue, relapse number, and disability

Mastery had no significant association with change in clinically significant fatigue from 2.5-year to 5-year review (Table 3). A positive trend was seen for resolution of fatigue, driven by those in the top quartile of mastery, but this attenuated and became nonsignificant on adjustment. Similarly, a reciprocal inverse trend was seen for developing fatigue but this disappeared on adjustment.

**Table 3:** Mastery and change in clinically significant fatigue, relapse number, and disability

Change in clinically significant fatigue								
	*n* (row %)		Loss of fatigue		*n* (row %)		Gain of fatigue	
	Always fatigued	Stops fatigue	aRR (95% CI)^a^	aRR (95% CI)^b^	Never fatigued	Starts fatigue	aRR (95% CI)^a^	aRR (95% CI)^b^
Mastery								
7–19	159 (87.4%)	23 (12.6%)	1.00 [Reference]	1.00 [Reference]	27 (69.2%)	12 (30.8%)	1.00 [Reference]	1.00 [Reference]
> 19–21	73 (79.4%)	19 (20.7%)	1.64 (0.95, 2.84)	1.61 (0.92, 2.84)	40 (78.4%)	11 (21.6%)	0.72 (0.36, 1.48)	0.82 (0.38, 1.74)
> 21–25	85 (86.7%)	13 (13.3%)	1.05 (0.56, 1.98)	0.83 (0.43, 1.61)	93 (86.9%)	14 (13.1%)	**0.43 (0.22, 0.85)**	0.55 (0.29, 1.05)
> 25–28	40 (72.7%)	15 (27.3%)	**1.96 (1.11, 3.47)**	1.54 (0.86, 2.76)	94 (81.7%)	21 (18.3%)	0.63 (0.34, 1.15)	0.76 (0.39, 1.47)
*Trend:*			*p* = *0.088*	*p* = *0.63*			*p* = *0.15*	*p* = *0.56*

Change in relapse number							
	*n* (row %)			Relapse decrease		Increase relapse	
	Relapse decrease	Stable relapse	Increase relapse	RR (95% CI)	aRR (95% CI)^c^	RR (95% CI)	aRR (95% CI)^c^
Mastery							
7–19	34 (15.3%)	163 (73.4%)	25 (11.3%)	1.00 [Reference]	1.00 [Reference]	1.00 [Reference]	1.00 [Reference]
> 19–21	13 (9.1%)	112 (78.3%)	18 (12.6%)	0.79 (0.43, 1.46)	0.91 (0.40, 2.06)	1.07 (0.61, 1.88)	1.00 (0.57, 1.74)
> 21–25	24 (11.4%)	165 (78.2%)	22 (10.4%)	0.96 (0.58, 1.59)	1.31 (0.69, 2.49)	0.93 (0.54, 1.59)	0.91 (0.52, 1.60)
> 25–28	17 (9.8%)	148 (85.6%)	8 (4.6%)	0.79 (0.45, 1.40)	1.19 (0.57, 2.50)	0.41 (0.19, 0.89)	0.53 (0.25, 1.12)
*Trend:*				*p* = 0.55	*p* = 0.57	*p* = 0.026	*p* = 0.25

Change in disability							
	*n* (row %)			Disability decrease		Increase disability	
	Disability decrease	Stable disability	Increase disability	aRR (95% CI)^a^	aRR (95% CI)^d^	aRR (95% CI)^a^	aRR (95% CI)^d^
Mastery							
7–19	12 (5.1%)	196 (83.1%)	28 (11.9%)	1.00 [Reference]	1.00 [Reference]	1.00 [Reference]	1.00 [Reference]
> 19–21	8 (5.1%)	132 (84.1%)	17 (10.8%)	0.96 (0.41, 2.26)	0.77 (0.35, 1.66)	0.90 (0.52, 1.58)	0.79 (0.45, 1.38)
> 21–25	10 (4.6%)	195 (88.6%)	15 (6.8%)	0.85 (0.38, 1.90)	0.57 (0.24, 1.35)	0.59 (0.33, 1.06)	0.56 (0.30, 1.06)
> 25–28	6 (3.4%)	160 (89.4%)	13 (7.3%)	0.61 (0.24, 1.58)	0.51 (0.20, 1.31)	0.69 (0.36, 1.31)	0.68 (0.34, 1.37)
*Trend:*				*p* = 0.30		*p *= 0.11	*p* = 0.32

All models by log-binomial regression, estimating risk ratio (RR) and adjusted risk ratios (aRR) (95% CI). Results in boldface denote statistical significance (p < 0.05)

^a^Multivariable log-binomial regression model adjusted for ongoing symptoms of recent relapse at each timepoint

^b^Multivariable log-binomial regression model adjusted for ongoing symptoms of recent relapse at each timepoint, and baseline age, sex, disability, treated comorbidity number, and depression risk

^c^Adjusted for baseline age, sex, disability, fatigue, treated comorbidity number, and depression risk

^d^Multivariable log-binomial regression model adjusted for ongoing symptoms of recent relapse at each timepoint, and baseline age, sex, fatigue, treated comorbidity number, and depression risk

In univariable models, those with 2.5-year mastery in the top quartile had 59% lower risk of increasing their diagnosed relapse number, though this association attenuated on adjustment. No association was seen for decrease in relapse number, however.

No associations were seen between 2.5-year baseline mastery and subsequent increase or decrease in disability.

## Discussion

We have shown that higher mastery is cross-sectionally associated with lower frequencies of depression risk, clinically significant fatigue, and severe disability, robust to adjustment, and prospectively with a lower risk of developing depression risk over 2.5 years’ follow-up. No prospective associations were seen for loss of depression risk, nor with any change in fatigue or disability, and no robust associations were seen for relapse number, either cross-sectionally or prospectively. These results suggest that developing a greater sense of mastery may prevent pwMS from developing depression, but associations with disability, relapse rate, and fatigue may reflect reverse causality.

Depression is a prevalent and debilitating symptom of MS with approximately 50% or more of pwMS experiencing a depressive disorder [20]. Our most important finding was that higher levels of mastery were associated with up to 90% reduced frequency of depression risk cross-sectionally and 60–70% lower risk of developing depression risk prospectively. These results are in accord with our earlier studies showing greater mastery was associated with significantly better mental health-related quality of life [8]. It is also consistent with other studies that demonstrated inverse associations in self-efficacy scores with depressions scores in pwMS [21, 22].

Depression in MS occurs more frequently than in the general population, potentially resulting from the same inflammatory aetiologic factors, and/or a consequence of the effects of MS and its clinical progression[23]. Regardless of its genesis, there is a potent role for stress in realising depression in pwMS, since peripheral inflammation can disrupt normal mood via the hypothalamic–pituitary–adrenal (HPA) axis, and likewise potentially the realisation of general inflammation due to stress through the same pathway [23]. This interaction provides a route by which stress reduction might lead to reduced depression risk. Our results are consistent with studies in both established MS [22, 24] and recently diagnosed MS [25], showing that persons of higher resilience have less depression and better quality of life. It is possible that persons of greater mastery are more resilient and thus, less at risk of becoming depressed due to the effects of their disease. It is also possible that persons who are more resilient may represent a particular population for whom the inflammatory effects of disease do not realise as much mood disruption via the HPA axis. Both these interpretations would align with the prospective finding that pwMS of higher mastery were less likely to develop depression risk. If, the resilience mechanism is valid, this represents a point of intervention for pwMS to reduce their risk of depression. Through mindfulness or other mental health interventions, resilience and mastery could be increased and could contribute to a reduced risk of developing depression.

Another possibility is that greater mastery could lead to an increased likelihood to engage in positive lifestyle behaviours like physical activity, cognitive training, or other social behaviours that could potentially realise the positive impacts on depression risk. Examination of these relationships would also be worthwhile.

Our findings for other clinical outcomes assessed are less indicative of a true association, being only present cross-sectionally for fatigue and disability but not prospectively. Previous studies have reported similar findings of an association between fatigue and mastery and related concepts in pwMS. For example, in a cross-sectional study of 139 pwMS, higher environmental mastery, the ability to create an environment suitable to one’s needs, assessed by a subdomain of the Ryff Happiness Scale [26], was inversely associated with fatigue severity [9]. For disability and mastery, less has been done. The aforementioned study by Schwartz and colleagues found no relationship between the Ryff Happiness Scale and disability as measured by EDSS [9]. Other prospective cohort studies of this relationship are yet needed to make definitive conclusions.

It is possible that increased resilience and less stress-associated inflammation could have a similar effect on relapse as seen for depression on risk of relapse. This is suggested by our data as those in the top quartile of mastery had a lower risk of increasing relapse number during follow-up; however, this was not significant after adjustment. That no associations were seen between mastery and relapse may be attributed to deficiencies in the measure. Participants were asked to report the number of doctor-diagnosed relapses they had experienced in the previous year. Such measures are unfortunately quite susceptible to recall bias and recall error, and a prospective measure of relapses would have been preferable. Thus, we are cautious in the interpretation of these results. There has been no investigations of mastery and relapse risk in MS so we have no ability to compare with the literature on this topic. Other studies of the mastery-relapse relationship should be undertaken, ideally prospective in nature so as to better assess causality of associations, if any.

## Strengths and limitations

Our study has many strengths, comprising a large international cohort of pwMS, utilising a prospective study design, and including a diversity of demographic, clinical, lifestyle, and outcome measures. The sample size gives us significant statistical power. We acknowledge appreciable attrition from our baseline survey, with 34.0% retention. This is to some extent a function of the healthy participant bias attendant to many longitudinal epidemiological studies, but may reflect the online nature of recruitment that can impact on loss of contact due to changed email addresses as well as a lack of a more personal contact that come from clinic-based studies. Given our exposure, mastery, and our outcomes of clinical severity, particularly depression, there is potential for attrition to impact on our observed results. That said, while we have lost some proportion of people with higher levels of depression, fatigue, and relapse rate, and lower mastery, that we continue to show a robust prospective relationship with mastery and subsequent depression risk may indicate the findings here underestimate a true effect. Assessments of these relationships in other samples, particularly with less attrition and attendant healthy participant bias, are worthwhile.

Other limitations include the self-reported nature of the survey, including MS diagnosis and MS phenotype, as well as clinical outcome measures. However, measures such as mastery and fatigue are necessarily subjective in nature, and validated tools were used wherever possible. Our prospective study design gives us the ability to assess causal directionality. The comprehensive data capture allows us to develop robust multivariable models that control for a great deal of potential confounding. Another potential limitation lies in not accounting for cognitive impairment as this might modulate the mastery-outcome relationships.

Our measure of depression only queried depression symptoms in the preceding two weeks, whereas it is possible that people may have developed and resolved depression symptoms in the 2.5-year period between reviews but before the two weeks before they completed the PHQ-9. Thus, a study seeking to validate these results might endeavour to seek additional modes of assessing depression, either more frequent survey or linkage with clinical data whereby greater ascertainment of depression symptoms could be made.

Finally, while mastery is a mode of assessing personal capacity to cope with stress and other issues, a related measure which we do not have measurement of is self-efficacy. Self-efficacy has been described as altering one’s expectations of mastery and success in a positive fashion to mental wellbeing [27]. While a related measure, mastery is distinct in being a function of the ability of the person to feel themselves in control of their lives [1], whereas self-efficacy includes elements of this, as well as social desirability, interpersonal competency, ego, and self-esteem [27]. While we believe that sense of self-control is important in coping for people with MS and thence to potentially have effects on aspects of mood and clinical progression, certainly for depression as an outcome, the other elements included in self-efficacy could be important as well. Moreover, mastery and self-efficacy would likely relate and so greater mastery could improve self-efficacy, as has been demonstrated in other settings [28]. Thus, a study seeking to replicate these results might include self-efficacy alongside measures of mastery.

## Conclusions

We have shown a strong positive association of higher mastery with less risk of depression, and a lower risk of developing depression in the future. Building mastery and resilience may thus represent a point of intervention to improve mental health in pwMS.

## Supplementary Information

Below is the link to the electronic supplementary material.

Supplementary file1 (DOCX 14 kb)

## Data Availability

Persons interested in acquiring the data underlying these analyses may contact Dr Sandra Neate or Dr Nupur Nag in regards and an anonymised data cut may be provided.

## Declarations

### Consenting process

Participants were first presented with a patient information sheet, at the end of which they indicated if they consented to participate. If they selected yes, they proceeded to the survey. If they selected no, the survey closed.

## References

[1] Pearlin LI, Schooler C (1978). The structure of coping. Journal of Health and Social Behavior.

[2] Bandura A (1977). Self-efficacy: Toward a unifying theory of behavioral change. Psychological Review.

[3] Mikula P, Nagyova I, Vitkova M, Szilasiova J (2018). Management of multiple sclerosis: The role of coping self-efficacy and self-esteem. Psychology, Health and Medicine.

[4] Riazi A, Thompson A, Hobart J (2004). Self-efficacy predicts self-reported health status in multiple sclerosis. Multiple Sclerosis.

[5] Krokavcova M, Nagyova I, van Dijk JP, Rosenberger J, Gavelova M, Middel B, Gdovinova Z, Groothoff JW (2008). Mastery, functional disability and perceived health status in patients with multiple sclerosis. European Journal of Neurology.

[6] Wassem R (1992). Self-efficacy as a predictor of adjustment to multiple sclerosis. Journal of Neuroscience Nursing.

[7] Motl RW, McAuley E, Snook EM (2007). Physical activity and quality of life in multiple sclerosis: Possible roles of social support, self-efficacy, and functional limitations. Rehabilitation Psychology.

[8] O'Kearney EL, Brown CR, Jelinek GA, Neate SL, Taylor KT, Bevens W, De Livera AM, Simpson S, Weiland TJ (2020). Mastery is associated with greater physical and mental health-related quality of life in two international cohorts of people with multiple sclerosis. Multiple Sclerosis and Related Disorders.

[9] Schwartz CE, Coulthard-Morris L, Zeng Q (1996). Psychosocial correlates of fatigue in multiple sclerosis. Archives of Physical Medicine and Rehabilitation.

[10] Hart S, Fonareva I, Merluzzi N, Mohr DC (2005). Treatment for depression and its relationship to improvement in quality of life and psychological well-being in multiple sclerosis patients. Quality of Life Research.

[11] Hadgkiss EJ, Jelinek GA, Weiland TJ, Pereira NG, Marck CH, Van Der Meer DM (2013). Methodology of an international study of people with multiple sclerosis recruited through web 2.0 platforms: Demographics, lifestyle, and disease characteristics. Neurology Research International.

[12] Weiland TJ, De Livera AM, Brown CR, Jelinek GA, Aitken Z, Simpson SL, Neate SL, Taylor KL, O’Kearney E, Bevens W, Marck CH (2018). Health outcomes and lifestyle in a sample of people with multiple sclerosis (HOLISM): Longitudinal and validation cohorts. Frontiers in Neurology.

[13] Howe LD, Hargreaves JR, Ploubidis GB, De Stavola BL, Huttly SR (2011). Subjective measures of socio-economic position and the wealth index: A comparative analysis. Health Policy and Planning.

[14] Craig CL, Marshall AJ, Sjöström M, Bauman AE, Booth ML, Ainsworth BE, Pratt M, Ekelund U, Yngve A, Sallis JF, Pekka OJA (2003). International physical activity questionnaire: 12-country reliability and validity. Medicine and Science in Sports and Exercise.

[15] Sangha O, Stucki G, Liang MH, Fossel AH, Katz JN (2003). The self-administered comorbidity questionnaire: A new method to assess comorbidity for clinical and health services research. Arthritis and Rheumatism.

[16] Krupp LB, LaRocca NG, Muir-Nash J, Steinberg AD (1989). The fatigue severity scale. Application to patients with multiple sclerosis and systemic lupus erythematosus. Archives of Neurology.

[17] Hohol MJ, Orav EJ, Weiner HL (1995). Disease steps in multiple sclerosis: A simple approach to evaluate disease progression. Neurology.

[18] Kroenke K, Spitzer RL, Williams JB (2001). The PHQ-9: Validity of a brief depression severity measure. Journal of General Internal Medicine.

[19] Simpson S, Taylor KL, Jelinek GA, De Livera AM, Brown CR, O'Kearney E, Neate SL, Bevens W, Weiland TJ (2019). Associations of demographic and clinical factors with depression over 2.5-years in an international prospective cohort of people living with MS. Multiple Sclerosis and Related Disorders.

[20] Patten SB, Marrie RA, Carta MG (2017). Depression in multiple sclerosis. International Review of Psychiatry.

[21] Tan-Kristanto S, Kiropoulos LA (2015). Resilience, self-efficacy, coping styles and depressive and anxiety symptoms in those newly diagnosed with multiple sclerosis. Psychology Health and Medicine.

[22] Berzins SA, Bulloch AG, Burton JM, Dobson KS, Fick GH, Patten SB (2017). Determinants and incidence of depression in multiple sclerosis: A prospective cohort study. Journal of Psychosomatic Research.

[23] Feinstein A, Magalhaes S, Richard JF, Audet B, Moore C (2014). The link between multiple sclerosis and depression. Nature Reviews Neurology.

[24] Koelmel E, Hughes AJ, Alschuler KN, Ehde DM (2017). Resilience mediates the longitudinal relationships between social support and mental health outcomes in multiple sclerosis. Archives of Physical Medicine and Rehabilitation.

[25] Tan-Kristanto S, Kiropoulos LA (2015). Resilience, self-efficacy, coping styles and depressive and anxiety symptoms in those newly diagnosed with multiple sclerosis. Psychology Health and Medicine.

[26] Ryff CD, Keyes CL (1995). The structure of psychological well-being revisited. Journal of Personality and Social Psychology.

[27] Sherer M, Maddux J (1982). The self-efficacy scale: Construction and validation. Psychological Reports.

[28] Warner LM, Stadler G, Lüscher J, Knoll N, Ochsner S, Hornung R, Scholz U (2018). Day-to-day mastery and self-efficacy changes during a smoking quit attempt: Two studies. The British Journal of Health Psychology.

