# Supplementary material for: Greater mastery is associated with lower depression risk in a large international cohort of people with multiple sclerosis over 2.5 years
Source: Qual Life Res. 2021 Nov 23;31(6):1789–98. doi: 10.1007/s11136-021-03033-7 (PMC9098535; doi:10.1007/s11136-021-03033-7)
Supplement: Supplementary file 1 — Supplementary file1 (DOCX 14 kb) [file 11136_2021_3033_MOESM1_ESM.docx]

Supplemental Table 1. Characteristics of depression risk, clinically significant fatigue, and relapse number at 2.5-year review.

| **Depression risk** |  |  |  |
| --- | --- | --- | --- |
|  | n/N (row %) | aPR (95% CI)^a^ | aPR (95% CI)^b^ |
| Mastery  7-19  >19-21  >21-25  >25-28  *Trend:* | 90/224 (40.2%)  27/149 (18.1%)  16/215 (7.4%)  7/174 (4.0%) | 1.00 [Reference]  **0.45 (0.31, 0.65)**  **0.19 (0.11, 0.30)**  **0.10 (0.05, 0.21)**  ***p<0.001*** | 1.00 [Reference]  **0.60 (0.41, 0.86)**  **0.31 (0.19, 0.52)**  **0.21 (0.10, 0.44)**  ***p<0.001*** |
| **Clinically significant fatigue** |  |  |  |
|  | n/N (row %) | aPR (95% CI)^a^ | aPR (95% CI)^c^ |
| Mastery  7-19  >19-21  >21-25  >25-28  *Trend:* | 188/227 (82.8%)  96/148 (64.9%)  100/213 (47.0%)  56/171 (32.8%) | 1.00 [Reference]  **0.78 (0.69, 0.89)**  **0.57 (0.49, 0.66)**  **0.40 (0.32, 0.49)**  ***p<0.001*** | 1.00 [Reference]  **0.87 (0.76, 1.00)**  **0.69 (0.59, 0.80)**  **0.50 (0.40, 0.62)**  ***p<0.001*** |
| Relapse number |  |  |  |
|  | n/N (col %) | IRR (95% CI) | aIRR (95% CI)^*^ |
| Mastery  7-19  >19-21  >21-25  >25-28  *Trend:* | 227/768 (29.6%)  151/768 (19.7%)  215/768 (28.0%)  175/768 (22.8%) | 1.00 [Reference]  **0.40 (0.26, 0.61)**  **0.47 (0.33, 0.68)**  **0.33 (0.21, 0.51)**  ***p<0.001*** | 1.00 [Reference]  **0.52 (0.32, 0.83)**  0.75 (0.50, 1.12)  0.68 (0.41, 1.13)  *p=0.074* |
| Depression risk and fatigue models by log-binomial regression, estimating adjusted prevalence ratio (aPR) (95% CI). Relapse number models by Poisson regression, estimating incidence rate ratio (IRR) (95% CI).  Results in boldface denote statistical significance (p<0.05).  a Adjusted for ongoing symptoms of relapse.  b Adjusted for ongoing symptoms of relapse, age, sex, disability, number of treated comorbidities, clinically significant fatigue, and prescription antidepressant medication use.  c Multivariable log-binomial model adjusted for ongoing symptoms of recent relapse, age, sex, disability, number of treated comorbidities, and depression risk.  d Multivariable models adjusted for age, sex, MS type, depression risk and clinically significant fatigue. | | | |

Supplemental Table 2. Characteristics of level of disability at 2.5-year review

|  | Mild | Moderate | Severe | Moderate vs mild | | Severe vs mild | |
| --- | --- | --- | --- | --- | --- | --- | --- |
|  | n (row %) | n (row %) | n (row %) | aPR (95% CI)^a^ | aPR (95% CI)^b^ | aPR (95% CI)^a^ | aPR (95% CI)^b^ |
| Mastery  7-19  >19-21  >21-25  >25-28  *Trend:* | 116 (49.2%)  92 (58.6%)  146 (66.4%)  132 (73.7%) | 76 (32.2%)  55 (35.0%)  57 (25.9%)  38 (21.2%) | 44 (18.6%)  10 (6.4%)  17 (7.7%)  9 (5.0%) | 1.00 [Reference]  0.94 (0.72, 1.24)  **0.71 (0.54, 0.94)**  **0.56 (0.41, 0.79)**  ***p<0.001*** | 1.00 [Reference]  1.06 (0.81, 1.40)  1.06 (0.80, 1.39)  0.91 (0.64, 1.28)  *p=0.73* | 1.00 [Reference]  **0.36 (0.19, 0.68)**  **0.38 (0.23, 0.63)**  **0.23 (0.12, 0.46)**  ***p<0.001*** | 1.00 [Reference]  **0.35 (0.18, 0.67)**  0.68 (0.40, 1.14)  **0.39 (0.18, 0.80)**  ***p=0.015*** |
| All models by log-binomial regression, estimating adjusted prevalence ratio (aPR) (95% CI). Results in boldface denote statistical significance.  a Adjusted for ongoing symptoms of relapse.  b Adjusted for ongoing symptoms of relapse, age, sex, clinically significant fatigue, number of treated comorbidities, and depression risk. | | | | | | | |
